# A Case Report of Aggressive Post-Infectious Hemophagocytic Lymphohistiocytosis in an Immunocompetent Adult

**DOI:** 10.7759/cureus.51334

**Published:** 2023-12-30

**Authors:** Nyein Wint Yee Theik

**Affiliations:** 1 Internal Medicine, Memorial Healthcare System, Pembroke Pines, USA

**Keywords:** timely treatment, diagnosis criteria, etoposide, hscore, hemophagocytic lymphohistiocytosis (hlh)

## Abstract

Hemophagocytic lymphohistiocytosis (HLH) is an acute inflammatory syndrome triggered by immune events such as infections, inflammation, autoimmune diseases, and malignancies. Initial presentations can range from vague symptoms to infectious features such as fever. Given its aggressive nature, timely diagnosis and immediate treatment are crucial to achieving optimal patient outcomes. Recently, the HLH score (HScore) criteria have been applied as diagnostic criteria, offering a broader scope compared to the previous HLH-2004 score, which was primarily based on pediatric populations. The standard treatment for decades has involved the combination of etoposide and high-dose steroids, and it is recommended to initiate treatment as soon as possible, even in the absence of a bone marrow test or when there is suspicion of the diagnosis. In this case presentation, we aim to underscore the significance of maintaining a high level of suspicion for HLH and the importance of promptly initiating treatment.

## Introduction

Hemophagocytic lymphohistiocytosis (HLH) is an acute inflammatory syndrome resulting from uncontrolled immune activation, primarily due to severe macrophage activation and the release of many inflammatory cytokines [1]. It is diagnosed less frequently than its actual occurrences, especially in adult populations [2]. A late diagnosis can occasionally lead to a poor prognosis and life-threatening situations. HLH can manifest either as a primary condition, known as familial, or sporadic, known as a secondary condition, commonly caused by infections, inflammation, malignancies, autoimmune diseases, and medications such as immune checkpoint inhibitors [3]. The most common infectious trigger is viral infections. Clinical features are nonspecific and often mimic infectious symptoms such as fever and multi-organ involvement [3].

The finding of hemophagocytosis in bone marrow biopsies is usually considered the standard label for the diagnosis of HLH [4]. The HLH-2004 diagnostic criteria were created based on the pediatric clinical trial and do not reflect to diagnose adult secondary HLH or distinguish adult HLH from inflammatory conditions such as sepsis and rheumatologic diseases [5]. The HLH score (HScore) is a newly developed risk stratification score in 2014 that can be applied reliably to diagnose HLH in pediatric and adult populations. The scoring system also helps guide further investigation and specific treatment plans [6]. HLH is commonly associated with a very high mortality rate regardless of etiology. Patients without treatment can have a rapid disease progression, leading to fatality within weeks or months from initial diagnosis [7]. Therefore, timely diagnosis is crucial in HLH to initiate specific treatment [8]. In this case report, we would like to discuss the patient with a severe aggressive disease course of idiopathic HLH who unfortunately passed away within a week of diagnosis.

## Case presentation

A 77-year-old Hispanic woman presented to the emergency department (ED) with fever, drenching sweats, generalized weakness, and unintentional weight loss for two weeks. The patient had a history of poorly controlled hypertension, hyperlipidemia, and gastroesophageal reflux disease (GERD). Her home medications included pantoprazole for GERD, metoprolol, amlodipine, hydrochlorothiazide, and enalapril for hypertension. A week before presenting to the ED, she visited an urgent care clinic for the same symptoms. Prior to the ED visit, she was diagnosed with a urinary tract infection caused by pan-sensitive *Escherichia coli*, although she did not exhibit urinary symptoms. She also took amoxicillin-clavulanate before admission, but her symptoms did not improve with antibiotics. The patient had no significant social history, such as smoking, heavy alcohol intake, or recreational drug use. Regarding family history, her father had passed away from coronary artery disease, but there were no other significant family health issues.

**Figure 1:**
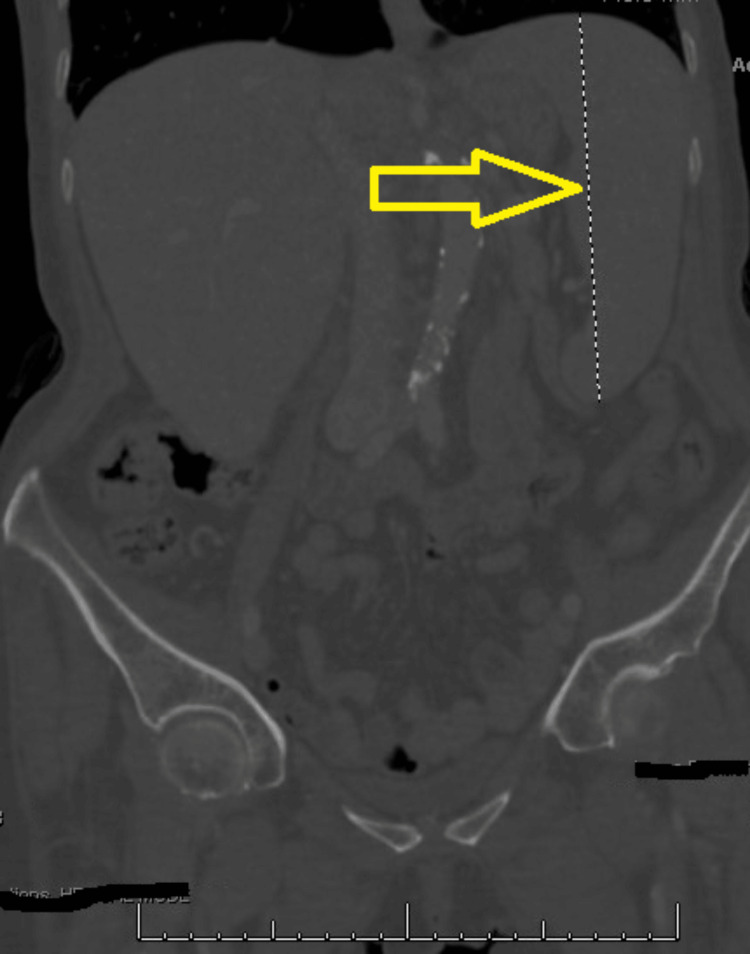
Splenomegaly on CT scan

Aside from a mild fever with a temperature of 38°C, she had normal blood pressure, a stable heart rate, and adequate oxygen levels in an initial assessment. Physical examination revealed signs of cachexia and dehydration, including dry mucus membranes. The abdominal examination showed hepatosplenomegaly, but other systemic examinations, including the cardiopulmonary assessment, were unremarkable. A complete blood count (CBC) revealed a decreased white cell count (WCC) of 2.1 x 10^3^/µL and a platelet count of 60 x 10^3^/µL. The comprehensive metabolic panel showed elevated liver function parameters, including total bilirubin and alkaline phosphatase. Right upper quadrant abdominal ultrasound indicated hepatomegaly with non-specific lymphadenopathy. Large splenomegaly was noted on abdominal and pelvic computed tomography (CT) scan (Figure 1).

The patient was initially prescribed broad-spectrum antibiotics to address the possibility of septic shock from the recent infection and fluid replacement. On her second day of admission, her WCC worsened despite antibiotics, and her hemoglobin dropped to 9.8 g/dL from the baseline. A biopsy was impossible due to the small size of the lymph nodes. Anemia workup included an iron panel, ferritin, vitamin B12, folate, and a hemolysis panel. The tests revealed an elevated ferritin level of 1790 ng/mL and signs of hemolysis, such as low haptoglobin and elevated lactate dehydrogenase. Blood leukemia-lymphoma phenotype analysis did not reveal abnormal blasts or aberrant antigenic expression. Fibrinogen levels decreased to 78 mg/dL, and triglycerides from the lipid panel elevated at 388 mg/dL. Autoimmune antibodies, used to rule out autoimmune hemolytic anemia, returned as unfavorable.

Considering the clinical manifestations and laboratory findings, an HScore was calculated to assess the possibility of HLH, resulting in a score of 234, indicating a 98-99% probability. Standard causative viral panels, such as Epstein Barr virus (EBV), cytomegalovirus, and Herpes tests, came back negative, along with sterile blood and urine cultures. On the third day of admission, the patient was treated with dexamethasone while scheduling biopsies, including a bone marrow and liver biopsy, to rule out malignancies as potential etiologies and confirm the diagnosis. The patient's condition was unstable, and intermittent febrile episodes, tachycardia, low blood pressure, and intermittent desaturation complicated her hospital course. Immediate biopsies could not be performed due to unstable vitals, and the patient declined to start etoposide despite it being recommended for immediate treatment initiation. The HScore diagnostic criteria comparison between day 1 and day 5 is shown in Table 1 [6].

**Table 1:** HScore diagnostic criteria comparison between day 1 and day 5 of admission Source: [6] AST, aspartate transaminase; BM, bone marrow

Metrics	Results	Scores	Patient results on Day 1	Patient results on Day 5
Known immunosuppression	No	+0	Present	Present
	Yes	+18		
Temperature (°C)	<38.4	+0		
	38.4-39.4	+33		
	>102.9	+49	Present	Present
Organomegaly	No	+0		
	Hepatomegaly or splenomegaly	+23		
	Hepatomegaly and splenomegaly	+38	Present	Present
Number of cytopenias	1 lineage	+0		
	2 lineages	+24	Present	
	3 lineages	+34		Present
Ferritin (ng/mL)	<2,000	+0	Present	
	2,000-6,000	+35		
	>6,000	+50		Present
Triglyceride (mg/dL)	<132.7	+0		
	132.7-354	+44		
	>354	+64	Present	Present
Fibrinogen (mg/dL)	>250	+0		
	<250	+30	Present	Present
AST (units/L)	<30	+0		
	>30	+19	Present	Present
Hemophagocytosis features on BM aspirate	No	+0		
	Yes	+35	Not performed	Not performed
Total Score			224	284

Follow-up blood work, such as CBC and comprehensive metabolic panels on the fourth and fifth days of admission, revealed worsening thrombocytopenia, liver function, and bilirubin levels. The patient also became acidotic, with a gradual decrease in bicarbonate levels. Unfortunately, the patient decompensated and died on the fifth admission day, and an autopsy could not be performed.

## Discussion

The HScore is a valuable tool for estimating the probability of HLH among suspected individuals. Notably, the probability of HLH is relatively low, less than 1%, for an HScore of less than or equal to 90 [6]. In contrast, an HScore of 250 or more is associated with a high probability of 99% [6]. We found that using a cutoff of 169 had 93% sensitivity and 86% specificity in diagnosing HLH [9]. To establish a diagnosis of HLH, patients must meet at least five out of nine HScore criteria.

Among the elderly population, common causes of HLH include viral infections, such as EBV, bacterial or fungal infections, certain malignancies such as T-cell lymphoma, and autoimmune diseases [10]. In our case, we ruled out active infections through blood culture and viral panels and malignancy through CT imaging. Given the aggressive nature and high mortality rate of HLH, immediate treatment, typically within a week, is recommended to improve patient outcomes [11]. The recommended treatment regimen consists of dexamethasone and etoposide, as treating with dexamethasone alone is considered inadequate [12].

Several predictive factors are associated with poor overall survival indicators in HLH patients. These factors include age over 45 years, low platelet counts, EBV association, and hyperferritinemia [13]. Our patient exhibited three factors: age over 45 years, initial presentation with thrombocytopenia, and hyperferritinemia, which unfortunately led to the patient's demise within five days of the initial diagnosis. The patient's refusal to commence etoposide treatment in combination with steroids may have also contributed to this unfortunate outcome.

## Conclusions

This case emphasizes the difficulties of diagnosing HLH and underscores its aggressive nature. Healthcare providers should maintain a high level of suspicion for HLH when they come across patients exhibiting compatible clinical features, even if classic symptoms or risk factors are absent. It is crucial to promptly initiate treatment upon suspicion of the diagnosis without waiting for results such as a bone marrow biopsy, which can be time-consuming and may lead to treatment delays. This proactive approach is vital in improving patient outcomes and preventing the progression of this life-threatening condition.
